# Uptake and correlates of cervical cancer screening among women attending a community-based multi-disease health campaign in Kenya

**DOI:** 10.1186/s12905-022-01702-4

**Published:** 2022-04-18

**Authors:** Yujung Choi, Saduma Ibrahim, Lawrence P. Park, Craig R. Cohen, Elizabeth A. Bukusi, Megan J. Huchko

**Affiliations:** 1 Duke Global Health Institute, Duke University, Durham, NC USA; 2 Kenya Medical Research Institute, Nairobi, Kenya; 3 Department of Obstetrics, Gynecology and Reproductive Sciences, Bixby Center for Global Reproductive Health, San Francisco, CA USA; 4 Department of Obstetrics and Gynecology, Duke University, Durham, NC USA

## Abstract

**Introduction:**

Despite the increased risk of cervical cancer among HIV-positive women, many HIV-care programs do not offer integrated cervical cancer screening. Incorporating self-collected Human Papillomavirus (HPV) testing into HIV programs is a potential strategy to identify women at higher risk for cervical cancer while leveraging the staffing, infrastructure and referral systems for existing services. Community-based HIV and HPV testing has been effective and efficient when offered in single-disease settings.

**Methods:**

This cross-sectional study was conducted within a community outreach and multi-disease screening campaigns organized by the Family AIDS Care and Education Services in Kisumu County, Kenya. In addition to HIV testing, the campaigns provided screening for TB, malaria, hypertension, diabetes, and referrals for voluntary medical male circumcision. After these services, women aged 25–65 were offered self-collected HPV testing. Rates and predictors of cervical cancer screening uptake and of HPV positivity were analyzed using tabular analysis and Fisher’s Exact Test. Logistic regression was performed to explore multivariate associations with screening uptake.

**Results:**

Among the 2016 women of screening age who attended the outreach campaigns, 749 women (35.6%) were screened, and 134 women (18.7%) were HPV-positive. In bivariate analysis, women who had no children (p < 0.01), who were not pregnant (p < 0.01), who were using contraceptives (p < 0.01), who had sex without using condoms (p < 0.05), and who were encouraged by a family member other than their spouse (p < 0.01), were more likely to undergo screening. On multivariable analysis, characteristics associated with higher screening uptake included: women aged 45–54 (OR 1.62, 95% CI 1.05–2.52) compared to women aged 25–34; no children (OR 1.65, 95% CI 1.06–2.56); and family support other than their spouse (OR 1.53, 95% CI 1.09–2.16). Women who were pregnant were 0.44 times (95% CI 0.25–0.76) less likely to get screened. Bivariate analyses with participant characteristics and HPV positivity found that women who screened HPV-positive were more likely to be HIV-positive (p < 0.001) and single (p < 0.001).

**Conclusions:**

The low screening uptake may be attributed to implementation challenges including long waiting times for service at the campaign and delays in procuring HPV test kits. However, given the potential benefits of integrating HPV testing into HIV outreach campaigns, these challenges should be examined to develop more effective multi-disease outreach interventions.

## Introduction

Women in sub-Saharan Africa face dual burdens of HIV and cervical cancer. Among the estimated 20.1 million girls and women living with HIV globally in 2020, almost three quarters were in sub-Saharan Africa [1]. Around ninety percent of the estimated 570,000 cervical cancer cases and 311,000 deaths in 2018 occurred among women living in low- and middle-income countries (LMICs) with sub-Saharan Africa having the greatest burden of disease [2]. In Kenya, cervical cancer remains the leading cause of cancer-related deaths and the second most common cancer among women [2].

Women living with HIV are at increased risk for infection with human papillomavirus (HPV), which is the causative agent in cervical cancer, which is an AIDS-defining disease [3]. International organizations have set ambitious targets to achieve both HIV and cervical cancer control. To combat the AIDS epidemic, UNAIDS set 90-90-90 targets for 2020, for countries to achieve 90% of individuals living with HIV knowing their status, 90% of individuals who test HIV positive being ART, and 90% of those achieving viral suppression [4]. Although there has been significant progress towards the target as reflected in the 2018 global estimates of 79-78-86 [5], challenges in achieving this target persist; recent studies have identified the need to continue providing peer-delivered linkage services, engaging in quality counseling, enhancing HIV education and treatment literacy, and reducing HIV stigma in community and among healthcare providers [6, 7]. The World Health Organization has set an analogous triad of targets to meet their plan for cervical cancer elimination: 90% of adolescents vaccinated, 70% of women appropriately screened with HPV and 90% treated by 2030 [8]. However, cervical cancer screening programs have been hindered by high attrition rates in LMICs primarily due to inadequate health care infrastructure to provide cervical cancer prevention services [9].

Cervical cancer and HIV screening, along with linkage to care, need to reach a wide swath of the population in order to achieve public health impact. The sizeable donor investment in HIV care has resulted in a marked improvement in related health care infrastructure [10, 11], in many places facilitating the integration of other health services, such as family planning and antenatal care. Cervical cancer screening in these settings has been most commonly offered through visual inspection with acetic acid (VIA) [12–14], requiring additional training, space and supplies. Screening through HPV testing could potentially simplify an integrated strategy [15–18], leveraging the existing laboratory and specimen tracking processes integral to HIV care while decreasing the need for clinical training to perform and interpret the VIA. Offering screening via self-collected specimens would remove the need for a pelvic exam, potentially allowing screening to be offered in outreach events and community settings where HIV testing campaigns have been successful [19, 20]. In fact, HPV testing offered through highly attended community health campaigns (CHCs) has been acceptable among women and can potentially reach a greater number of women compared to clinic-based screening [21]. To our knowledge, self-collected HPV testing integrated into CHCs offering HIV along with screening for other diseases has not been evaluated.

To address the cervical cancer screening gap in Kenya, we modified an HPV-based cervical cancer screening model offered through CHCs in the Nyanza region of western Kenya, an area with high rates of HIV [22, 23]. We leveraged the Family AIDS Care and Education Services’ (FACES) [24] planned CHCs offering multi-disease testing to achieve a high population coverage for HIV-testing and linkage to care for both HIV and cervical cancer through CHCs in western Kenya. The overall description of the multi-disease campaign is discussed in another publication [25, 26]. In this paper, we describe the acceptability and uptake of a model of integrated HPV-based cervical cancer screening as part of a series of multi-disease community health campaigns offered in Kisumu, Kenya. Further, we sought to describe the prevalence and predictors of both screening and positive HPV results among women attending these campaigns.

## Methods

### Study design

**Fig. 1:**
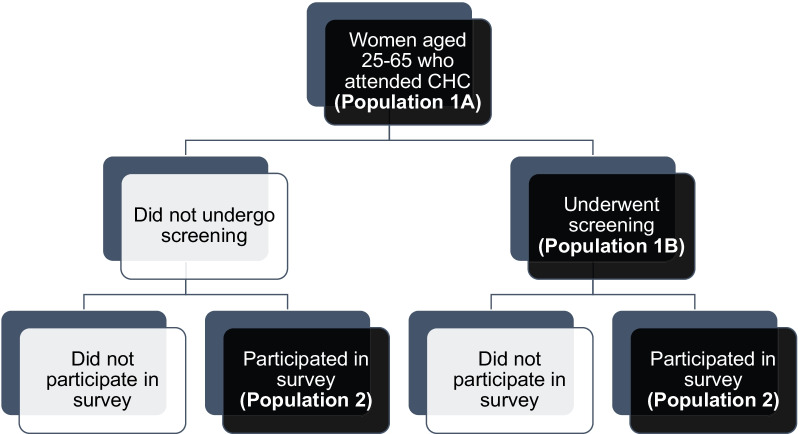
Study population flowchart

This was a cross-sectional study of participants attending a multi-disease community health campaign. The overall population at the campaign consisted of residents of all ages from four peri-urban sub-counties in Kisumu County. At the health campaign, women between the ages 25 and 65, were invited to screen for cervical cancer with self-collection HPV testing (Study Population 1A) (see Fig. 1). As participants were leaving the campaign area, we consecutively approached women in this age group to participate in a survey of knowledge and attitude towards cervical cancer and screening in women, with a goal of equal number of women who participated in screening and did not undergo screening (Study Population 2).

### Study procedures

**Fig. 2:**
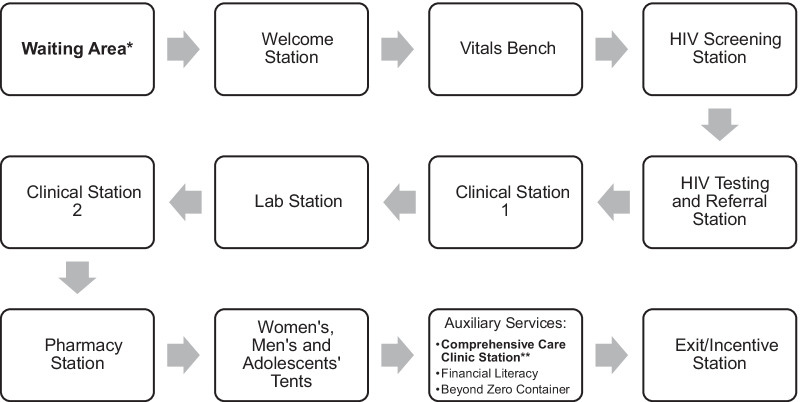
Flow diagram of the multi-disease health campaign. *At this station, group education on cervical cancer as well as HIV, diabetes, hypertension, tuberculosis, malaria, sexually transmitted infections screening, pregnancy testing, and voluntary medical male circumcision was provided to ensure participants are fully informed about the tests and services offered at the health campaign. The topics covered in cervical cancer education included: causes of cervical cancer, signs and symptoms of cervical cancer, prevention strategies, screening methods such as self-collection HPV testing, and information about follow up care with positive HPV test results. **Three study activities related to cervical cancer were conduced at this station: (1) additional group education about cervical cancer and cervical cancer screening (this education consisted of a short description of the anatomy of the cervix, symptoms of cervical cancer and pre-cancer, risk factors of cervical cancer such as positive HIV status, ways to decrease cervical cancer risk (such as having fewer sexual partners and having sex at an older age), prevention strategies, eligibility for cervical cancer screening, misconceptions about cervical cancer (such as association between cervical cancer risk and poor hygiene or infertility), treatment services in local health facilities, and instructions of self-collection HPV testing); (2) HPV self-sampling collection by participants; and (3) administration of survey

Between April and June 2018, we offered HPV-based cervical cancer screening as part of multi-disease CHCs run by the FACES program in four communities in the informal settlement area of Obunga a slum in the northwestern part of Kisumu City. As described in detail elsewhere [25, 26], all residents in the study communities were enumerated through door-to-door home visits by the study and program teams prior to the campaigns, where information about dates and services was provided. For the duration of three months, the following health services were routinely provided at the CHCs: HIV testing and counseling with referrals as needed; screening and referral for tuberculosis, malaria, hypertension, diabetes, and cervical cancer; and referrals for voluntary male medical circumcision (see Fig. 2). Families were encouraged to attend together [26].

At each CHC, female participants aged 25–65 were invited to attend a group education module on HPV and cervical cancer, covering the following topics: the anatomy of the cervix; symptoms of cervical cancer and pre-cancer; risk factors of cervical cancer such as positive HIV status, increased number of sexual partners, and young age at first sexual debut; prevention strategies; eligibility criteria for cervical cancer screening; misconceptions about cervical cancer such as association between cervical cancer risk and poor hygiene or infertility; and treatment services offered in local health facilities. Participants also received instructions on self-collection HPV testing using pictorial diagrams, after which they were offered cervical cancer screening. Women who decided to screen were asked to provide basic demographic information for follow-up, given a collection vial, and then shown a partitioned, private area within the tent designated for cervical cancer screening. After submitting their specimen, women participated in a brief post-screening survey about their experience with self-collection and preference for HPV test result notification (text message, phone call or home visit). Upon leaving the campaign tent, we sought to interview approximately 150 women who accepted screening and 150 who declined to undergo screening to ascertain factors related to their decision to screen. Data were collected using Open Data Kit [27], an open-source, mobile platform for collecting, managing, and using data in resource-limited settings. These data included demographics, basic health information, prior cervical cancer screening, prior HIV testing, self-reported HIV status, sexual risk behavior, and use of contraceptives.

Specimens were stored at room temperature until processing using the *care*HPV test system (Qiagen, Germantown, MD, USA), which was done in batches of ninety within two weeks of collection per the manufacturers’ guidelines. HPV test results were provided by the cervical cancer screening program staff using the women’s preferred method of notification. Women were given follow-up instructions based on the Kenya Ministry of Health guidelines. Those who tested negative for HPV were recommended to repeat screening in five years. Women who were HPV negative, but tested positive for HIV at the campaign, or were known to be HIV positive, were instructed to repeat cervical cancer screening in one year. All women who tested positive for HPV, regardless of HIV status, were referred to one of three sub-county hospitals within a ten-kilometer radius for cryotherapy treatment, which was provided at no cost. Results of this follow-up strategy and treatment uptake have been previously published elsewhere [28].

### Data analysis

There were three populations from which the data were collected for this study (see Fig. 1). First, at the CHCs, all women (Study Population 1A) completed a structured interviewer-administered questionnaire on sociodemographic characteristics, clinical characteristics, and reproductive characteristics (age of first sexual intercourse, parity, use of contraceptives, prior HIV testing, and self-reported HIV serostatus). We abstracted data from all women 25–65 years old (and therefore potentially eligible to screen) to compare the facilitators of screening uptake between those who screened and those who declined to be screened. Among the sub-population of women who screened (Study Population 1B), we used these baseline questionnaires to evaluate the predictors of HPV positivity among those who screened, with comparisons made between those who screened positive and those who screened negative. The last population (Study Population 2) was comprised of consecutively sampled women leaving the campaign tent, to identify an equal number who screened and chose not to be screened. These women were asked to complete a survey to assess their motivation for screening and knowledge of cervical cancer and screening. The knowledge section of the survey comprised of five sets of two statements (one fact and one common misconception about cervical cancer, HPV, or risk factors), of which participants selected one statement as true. One point was given for each correctly identified fact and zero points were given for incorrect answers or no response. A composite score was created by averaging the ten items.

Descriptive statistics were used to report screening uptake and prevalence of HPV positivity in this population. Chi-squared and Fisher’s Exact tests were used to compare the association of categorical exploratory variables of cervical cancer screening with screening outcomes while Student’s t-test was used to compare means of continuous predictor variables by screening outcomes. Additionally, we explored the association of demographic and clinical variables, including HIV status, antiretroviral therapy, and contraceptive use, as well as HIV sexual transmission risk behavior with screening outcome using logistic regression. Those variables with p-values less than 0.1 at the bivariable level were included in a multivariable logistic regression model for controlling potential confounding effect. In the multivariable analysis, variables with p-value < 0.05 were considered as associated factors. All analyses were performed using Stata/MP version 17 (StataCorp). As previously mentioned, the analysis for linkage-to-treatment for women who tested positive for HPV is reported elsewhere [28].

This study obtained ethics approval from Kenya Medical Research Institute Ethical Review Committee (KEMRI SERU No. SSC 2918), Institutional Review Boards of Duke University (IRB Pro00085971) and the University of California, San Francisco. Women provided written informed consent prior to participating in any study activity such as undergoing cervical cancer screening or completing a survey.

## Results

### CHC attendance and determinants of cervical cancer screening uptake and HPV positivity

A total of 2106 women between 25 and 65 years old attended CHCs during 22 days of campaigns across four communities (Study Population 1A). Among these, 749 (35.6%) participated in HPV self-testing (Population 1B). More than half (58.8%) of the screen-eligible women who attended the CHCs were between 25 and 34 years and most (88.5%) were either currently or previously married (Table 1). Nearly one-third of the population (30.3%) had no primary school education or had not completed primary school education while the majority (69.5%) earned income. At the time of the study, 410 women (19.5%) had been diagnosed with HIV, of whom 396 (97.8%) were on ART.

**Table 1:** Characteristics of participants by cervical cancer screening uptake, bivariate and multivariable models

Variables	All n = 2106	Screened n = 749	Not screened n = 1357	p-value	Adjusted odds ratio of screening (95% CI)	p-value
Age, n (%)				0.107		
25–34 years	1238 (58.8)	430 (57.4)	808 (59.5)		1.0	
35–44	492 (23.4)	186 (24.8)	306 (22.6)		1.29 (0.98–1.71)	0.071
45–54	221 (10.5)	88 (11.8)	133 (9.8)		1.62 (1.05–2.52)	0.030
55–65	155 (7.4)	45 (6.0)	110 (8.1)		1.28 (0.59–2.78)	0.535
Relationship status, n (%)				0.167	*	N/A
Single	235 (11.6)	98 (13.3)	137 (10.6)			
Married	1454 (71.6)	513 (69.7)	941 (72.6)			
Widowed, divorced, or separated	343 (16.9)	125 (17.0)	218 (16.8)			
Highest education level completed, n (%)				0.585	*	
None or some primary	615 (30.3)	225 (30.5)	390 (30.1)			
Primary	650 (32.0)	224 (30.4)	426 (32.9)			
Some secondary	337 (16.6)	126 (17.1)	211 (16.3)			
Secondary school	275 (13.5)	99 (13.4)	176 (13.6)			
Beyond secondary	155 (7.6)	64 (8.7)	91 (7.0)			
Employed, n (%)	1413 (69.5)	529 (71.7)	884 (68.2)	0.109	*	N/A
Distance to health campaign (mean km ± SD)	2.7 (10.7)	3.2 (13.9)	2.2 (6.0)	0.398	*	N/A
Polygamy, n (%)	167 (11.5)	65 (12.7)	102 (10.8)	0.303	*	N/A
HIV Status, n (%)				0.104	*	
Positive	410 (22.0)	134 (19.9)	276 (23.1)			
Negative	1457 (78.0)	540 (80.1)	917 (76.9)			
ART	396 (97.8)	132 (98.5)	264 (97.4)	0.724	*	N/A
Number of livebirths, n (%)				0.002		
0	91 (4.9)	48 (6.7)	43 (3.8)		1.65 (1.06–2.56)	0.026
1	240 (12.9)	95 (13.2)	145 (12.7)		0.97 (0.72–1.31)	0.839
2	464 (24.9)	180 (25.1)	284 (24.8)		0.94 (0.73–1.20)	0.603
3	416 (22.3)	132 (18.4)	284 (24.8)		0.69 (0.58–0.89)	0.005
> 3	652 (35.0)	263 (36.6)	389 (34.0)		1.0	
Age at first sex, n (%)				0.578	*	N/A
10–14	238 (12.3)	80 (11.1)	158 (13.1)			
15–19	1442 (74.8)	549 (76.5)	893 (73.7)			
20–24	220 (11.4)	79 (11.0)	141 (11.6)			
25–30	29 (1.5)	10 (1.4)	19 (1.6)			
Number of sexual partners in the last 3 months, n (%)				0.264	*	N/A
0	344 (17.8)	118 (16.4)	226 (18.7)			
1	1516 (78.6)	579 (80.6)	937 (77.4)			
2	48 (2.5)	13 (1.8)	35 (2.9)			
≥ 3	21 (1.1)	8 (1.1)	13 (1.1)			
Sex without condom in the last 3 months, n (%)	1080 (65.6)	421 (68.8)	659 (63.7)	0.036	1.15 (0.90–1.47)	0.261
Pregnant, n (%)	87 (4.9)	19 (2.7)	68 (6.3)	0.001	0.44 (0.25–0.76)	0.004
Contraceptive use in the last 12 months	1723 (97.1)	669 (98.7)	1054 (96.2)	0.002	Omitted due to collinearity	
Who encouraged participant to attend campaign				0.001		
Husband	90 (4.7)	24 (3.3)	66 (5.5)		0.67 (0.38–1.17)	0.157
Another family member	299 (15.5)	141 (19.6)	158 (13.1)		1.53 (1.09–2.16)	0.014
Friend or neighbor	587 (30.4)	208 (29.0)	379 (31.3)		1.0	
Local councilor and religious leader	387 (20.1)	147 (20.5)	240 (19.8)		1.08 (0.79–1.47)	0.642
Other	566 (29.3)	198 (27.6)	368 (30.4)		0.99 (0.75–1.32)	0.968

*Not included in the multivariable model

On bivariate analysis comparing the characteristics of women who completed or did not complete cervical cancer screening (Study Population 1B), women who had no children (6.7% vs. 3.8%; p = 0.002) and women who were not pregnant at the time of the campaign (97.3% vs. 93.7%; p = 0.001) were more likely to undergo screening (Table 1). Women who were using contraceptives in the last 12 months (98.7% vs. 93.7%; p = 0.002), who had sex without using condoms in the last three months (68.8% vs. 63.7%; p = 0.036), and who were encouraged by a family member other than their spouse (19.6% vs. 13.1%; p = 0.001), were also more likely to participate in screening. Age, relationship status, education level, HIV status, and distance to the health campaign were not associated with HPV screening uptake.

In the multivariate model of cervical cancer screening uptake (adjusted for age, number of children, sex without a condom, current pregnancy, and external social support), women aged 45–54 were 1.62 times (95% CI 1.05–2.52) more likely to undergo screening than women aged 25–34 years. Women who did not have any children and who were encouraged to attend CHC by another family member other than their spouse were 1.65 (95% CI. 1.06–2.56) and 1.53 times (95% CI 1.09–2.16) more likely, respectively, to undergo screening than those in the referent group. Those who were pregnant at the time of the CHC were 0.44 times (95% CI 0.25–0.76) less likely to get screened. Of the 749 women who underwent screening, 18.7% screened positive for HPV. Women who screened HPV-positive were more likely to be HIV-positive (p < 0.001) and single (p < 0.001) (Table 2).

**Table 2:** Sociodemographic and clinical characteristics of participants by HPV positivity

Variables	All n = 749	HPV positive n = 140	HPV negative n = 609	p-value
Age (mean years ± SD)	35.7 (9.6)	34.8 (9.3)	35.9 (9.6)	0.362
Age, n (%)				0.348
25–34 years	430 (57.4)	89 (20.7)	341 (79.3)	
35–44	186 (24.8)	28 (15.1)	158 (84.9)	
45–54	88 (11.8)	14 (15.9)	74 (84.1)	
55–65	45 (6.0)	9 (20.0)	36 (80.0)	
Relationship status, n (%)				< 0.001
Single	98 (13.3)	35 (35.7)	63 (64.3)	
Married	513 (69.7)	75 (14.6)	438 (85.3)	
Widowed, divorced, or separated	125 (17.0)	26 (20.8)	99 (79.2)	
Highest education level completed, n (%)				0.806
None or some primary	225 (30.5)	38 (16.9)	187 (83.1)	
Primary	224 (30.4)	44 (19.6)	180 (80.4)	
Some secondary	126 (17.1)	25 (19.8)	101 (80.2)	
Secondary school	99 (13.4)	16 (16.2)	83 (83.8)	
Beyond secondary	64 (8.7)	14 (21.9)	50 (78.1)	
Employed, n (%)				0.600
Yes	529 (71.7)	95 (18.0)	434 (82.0)	
No	209 (28.3)	41 (19.6)	168 (80.4)	
Polygamy, n (%)	65 (12.7)	10 (15.4)	55 (84.6)	0.851
HIV status, n (%)				< 0.001
Positive	134 (19.9)	44 (32.8)	90 (67.2)	
Negative	540 (80.1)	82 (15.2)	458 (84.8)	
ART, n (%)	132 (98.5)	43 (32.6)	89 (67.4)	0.551
Number of livebirths, n (%)				0.200
0	48 (6.7)	13 (27.0)	35 (73.0)	
1	95 (13.2)	22 (23.2)	73 (76.8)	
2	180 (25.1)	33 (18.3)	147 (81.7)	
3	132 (18.4)	18 (13.6)	114 (86.4)	
> 3	263 (36.6)	46 (17.5)	217 (82.5)	
Age at first sex, n (%)				0.420
10–14	80 (11.1)	10 (12.5)	70 (87.5)	
15–19	549 (76.5)	106 (19.3)	443 (80.7)	
20–24	79 (11.0)	13 (16.5)	66 (83.5)	
25–30	10 (1.4)	1 (10.0)	9 (90.0)	
Number of sexual partners in the last 3 months, n (%)				0.305
0	118 (16.4)	27 (22.8)	91 (77.2)	
1	579 (80.6)	98 (16.9)	481 (83.1)	
2	13 (1.8)	3 (23.1)	10 (76.9)	
≥ 3	8 (1.1)	2 (25.0)	6 (75.0)	
Sex without condom in the last 3 months, n (%)	421 (68.8)	72 (17.1)	349 (82.9)	0.647

The total column (all participants who underwent screening) shows column percentages whereas the rest of the columns (HPV positive and HPV negative) are row percentages

### Knowledge and attitudes towards cervical cancer and screening

Three hundred and twenty-three women completed the additional survey on knowledge and attitude towards cervical cancer and factors that contribute to cervical cancer screening (Study Population 2). Seventy eight percent participated in the education module on HPV and cervical cancer during the health campaign, which did not differ by screening status (Table 3). Among these women, 98% self-reported that they understood the cervical cancer education provided at the CHCs. There was no difference in composite knowledge scores based on the knowledge statements between women who underwent screening and those who did not (3.92 vs. 3.97; p = 0.696). In addition to the knowledge questions, women were asked to identify the next step for treatment after a positive HPV test, and 93.7% of the women who completed screening were able to correctly recall cryotherapy as the next step.

**Table 3:** Knowledge and attitudes of cervical cancer and screening among interviewed participants by screening status

Variables	All n = 323	Screened n = 157	Not screened n = 166	p-value
Participated in education module on HPV and cervical cancer at CHC, n (%)	252 (78.0)	125 (79.6)	127 (76.5)	0.505
Understood education about cervical cancer provided at CHC, n (%)	247 (98.0)	121 (96.8)	126 (99.2)	0.211
Composite score of knowledge assessment survey*, mean (SD)	3.94 (1.21)	3.92 (1.22)	3.97 (1.19)	0.696
Knowledge of HPV and Cervical Cancer**				0.464
Women who have HPV are at higher risk for developing cervical cancer in the future, but do not have cervical cancer now, n (%)	266 (82.4)	127 (80.9)	139 (83.7)	
Having HPV means a woman has cervical cancer, n (%)	45 (13.9)	22 (14.0)	23 (13.9)	
Did not respond, n (%)	12 (3.7)	8 (5.1)	4 (2.4)	
Knowledge of HPV and Fertility**				0.023
Testing positive for HPV does not mean I cannot bear children	238 (73.7)	110 (70.1)	128 (77.1)	
If I test positive for HPV, I cannot bear children, n (%)	54 (16.7)	35 (22.3)	19 (11.5)	
Did not respond, n (%)	31 (9.6)	12 (7.6)	19 (11.5)	
Knowledge of Risk Factors about Family Planning**				0.719
Family planning methods do not increase women’s risk for HPV, n (%)	227 (70.3)	108 (68.8)	119 (71.7)	
Family planning methods increase women’s risk for HPV, n (%)	52 (16.1)	28 (17.8)	24 (14.5)	
Did not respond, n (%)	44 (13.6)	21 (13.4)	23 (13.9)	
Knowledge of Risk Factors about Condom Use**				0.082
If I test HPV negative I can prevent infection by using a condom, n (%)	259 (80.2)	134 (83.4)	125 (75.3)	
If I test HPV negative I can prevent infection by washing, n (%)	38 (11.8)	14 (8.9)	24 (14.5)	
Did not respond, n (%)	26 (8.1)	9 (5.7)	17 (10.2)	
Knowledge of Local Healthcare Facilities**				< 0.001
There are places in Kisumu where I can get a safe and easy treatment for HPV if I test positive, n (%)	284 (87.9)	136 (86.6)	148 (89.2)	
There is no treatment if I test positive for HPV, n (%)	23 (7.1)	19 (12.1)	4 (2.4)	
Did not respond, n (%)	16 (5.0)	2 (1.3)	14 (8.4)	
Prefer to have a cervical cancer-specific campaign, n (%)	214 (66.3)	90 (57.3)	124 (74.7)	0.001

*Score = 0 if participant answered incorrectly or did not know the answer; score = 1 if participant answered correctly

**Participants were asked to identify a true statement out of the two statements listed per topic

A larger proportion of women who did not complete cervical cancer screening at the CHCs compared to those who did (74.7% vs. 57.3%; p = 0.001) indicated that they would prefer to have a cervical cancer-specific campaign rather than an integrated multi-disease health campaign. Among women who preferred a multi-disease campaign, regardless of their cervical cancer screening status, 73.4% mentioned the ability to access multiple testing and treatments as the primary reason for their preference.

### Intentions and motivations for cervical cancer screening

Among survey participants, older women were more likely to undergo screening (37.7 years vs. 34.8 years; p = 0.009) (Table 4). The majority of women in both screening groups reported learning about health in general as the primary reason for attending the CHC. Among women who attended the CHC, the opportunity for cervical cancer screening (33.1%) was the second most commonly cited reason for attendance. There was no difference in the average number of services in addition to cervical cancer screening that women accessed at the CHC between women who underwent cervical cancer screening and those who did not. On average, women from both screening groups attended approximately two additional services, the most common being HIV testing and hypertension screening. Less than half (47.0%) stated that they had ever been screened for cervical cancer prior to the campaign.

**Table 4:** Intentions and motivations for screening among interviewed participants by screening status

Variables	All n = 323	Screened n = 157	Not screened n = 166	p-value
Age (mean years ± SD)	36.7 (10.0)	37.7 (10.6)	34.8 (9.2)	0.009
Age, n (%)				0.017
25–34 years	170 (52.6)	75 (47.8)	95 (57.2)	
35–44	92 (28.5)	42 (26.8)	50 (30.1)	
45–54	41 (12.7)	29 (18.5)	12 (7.2)	
55–65	20 (6.2)	11 (7.0)	9 (5.4)	
Reasons for attending CHC				< 0.001
Cervical cancer screening	57 (17.7)	52 (33.1)	5 (3.0)	
HIV testing	19 (5.9)	7 (4.5)	12 (7.2)	
Family planning	14 (4.3)	3 (1.9)	11 (6.6)	
Learn about health	169 (52.3)	79 (50.3)	90 (54.2)	
Other*	64 (19.8)	16 (10.2)	48 (28.9)	
Number of other services accessed at campaign besides CCS, mean (SD)	1.94 (1.1)	1.96 (1.1)	1.92 (1.0)	0.778
Had prior cervical cancer screening, n (%)	116 (47.0)	45 (30.6)	71 (71.0)	< 0.001
Did not know CCS offered	120 (37.2)	58 (36.9)	62 (37.4)	1.000
Knew CCS offered	203 (62.9)	99 (63.1)	104 (62.7)	
Knew CCS offered and planned to screen	92 (45.3)	69 (69.7)	23 (22.1)	< 0.001
Knew CCS offered, but did not plan to screen	111 (54.7)	30 (30.3)	81 (77.9)	
How participants heard about CCS prior to campaign if they knew CCS was offered				0.186
Friends/family	43 (21.2)	23 (23.2)	20 (19.2)	
Written materials	27 (13.3)	16 (16.2)	11 (10.6)	
Home visit	43 (21.2)	24 (24.2)	19 (18.3)	
Publicizing within community	81 (39.9)	31 (31.3)	50 (48.1)	
Other	9 (4.3)	5 (5.1)	4 (3.9)	

*This category includes screening for malaria, tuberculosis, hypertension, and diabetes

Almost two thirds of women surveyed had been aware that cervical cancer screening was going to be offered at the CHC, with no difference between screening groups (63.1% of screeners vs 62.7% of non-screeners). The majority of women who knew about screening had made their decision prior to coming to the campaign, and the majority proceeded with that decision (69.7% of screeners planned to screen, while 77.9% of non-screeners had not intended to screen). The primary reason for getting screened was to know their HPV status, which was true even among those who had known about the availability of cervical cancer screening at the campaign and had no intention to get screened, but ultimately decided to get screened.

Among those who did not undergo screening, 77.9% had known that cervical cancer was going to be offered at the campaign and had not intended to undergo screening. The primary reason for not participating in HPV screening was reporting having undergone cervical cancer screening prior to the CHC. Lastly, very few of the non-screeners listed campaign or procedure-related reasons for not screening such as lack of time, not having sufficient privacy, complex or confusing self-sampling instruction, fear of self-collection, and discomfort or pain with self-collection.

## Discussion

The goal of this study was to assess the acceptability and effectiveness of HPV-based cervical cancer screening on the part of women during a multi-disease screening campaign by looking in depth at factors related to screeners’ and non-screeners’ participation. Overall, participation in this screening among women who were taking part in the campaign (35.6%) already was lower than anticipated. This lower percentage may be attributable to a higher than anticipated baseline rate of screening (47%) in the study communities based upon the subset of women who completed the survey. Within this population, socioeconomic factors such as education level, relationship status, occupation, and HIV status did not affect the women’s decision to undergo cervical cancer screening.

Despite the fact that there were six health services offered through the multi-disease campaign, non-screeners elected to receive an average of only two of the health services (HIV testing and hypertension screening), while screeners chose to receive an average of only three (HIV testing, hypertension screening, and cervical cancer screening). At the same time, although the cervical cancer education module provided at the CHCs was associated with women’s increased knowledge about cervical cancer, greater knowledge itself was not associated with an increase in screening. However, our findings from a multivariable analysis did show that women with fewer children were more likely to be screened. These findings lead us to suggest that the greatest barrier to cervical cancer screening in multi-disease campaigns may be largely attributable to logistical reasons rather than factors related to the procedure for self-sampling HPV testing, such as fear, discomfort, or pain associated with collecting the HPV specimen. Several previous studies have reported challenges in multi-disease health campaigns of maintaining efficiency and providing quality services to a large population in a short period of time [29]. The nature of large-scale health campaigns renders long wait times inevitable, which discourages women with children from being screened. To overcome such logistical barriers, systemic challenges that hinder optimal care should be identified and addressed.

In our study, women who had used contraceptives in the last 12 months were also more likely to undergo screening than those who had not. The implication of this finding is that women who use contraceptives are more familiarity with reproductive health services and are more informed about their reproductive health.

Our multivariable analysis showed that women between the ages of 45 and 54 were more likely, to be screened at the campaign compared to those between the ages of 25 and 34. This finding may be attributable to the fact that older women may have participated in more health services and learned about cervical cancer and the importance of screening, which motivated them to undergo screening. However, the analysis also showed a low rate of screening for those in the oldest age group, between 54 and 65. This finding is contrast to other studies that have observed higher screening rates in older, more educated, wealthier women, and in those living in urban areas who were more likely to have had experience with the health system for a longer period of time and, therefore, were more likely to have participated in a cervical cancer screening [30, 31]. One possible explanation for our findings is that older women may have had more opportunities to be screened previously and therefore, chose not to do so at this campaign. Unfortunately, our study did not examine when women were screened for cervical cancer last and thus cannot confirm this conclusion. Alternatively, it may be related to discomfort with screening in the semi-public environment of the CHC. Nonetheless, one study based in Kenya found that older women aged 35–49 were more likely than younger women to have received screening at some time, but were less likely to have up-to-date screening (screening within the last year) [32]. This highlights the much-needed cervical cancer prevention efforts for older women who are at higher risk of developing cervical cancer, particularly those who are also living with HIV and are recommended to have cervical cancer screening annually.

Women whom a family member other than their spouse encouraged to attend the campaign were more likely to be screened for cervical cancer. Other studies have found that married women were more likely to undergo screening than those who were unmarried, for such reasons as having their spouse’s social and financial support [33, 34]. Our study, however, demonstrated the value of emotional support from other female members in the household or from having heard about the screening process from a neighbor. This finding highlights a need to focus interventions on educating men who can help encourage positive health-seeking behavior. Several studies have called for educational interventions that target men to teach them about the causes of cervical cancer, promote their support for screening and treatment, and allay their misconceptions about the disease [35, 36]. One study in Uganda found that a male partner’s involvement increased follow‐up significantly among women who were referred for a colposcopy after a letter was sent that asked the husbands to accompany their spouse to the treatment [37]. Educational interventions, and the resulting increased spousal support, will likely lead more women to have cervical cancer screenings.

In this study cervical cancer screening uptake did not differ statistically significantly between women who had tested positive and negative for HIV before they attended the campaign. This was unexpected, as our education module informed women that HIV is a risk factor for cervical cancer and that annual cervical cancer screening is recommended for women living with HIV. It is possible that our participants with HIV had been screened for cervical cancer already at their ART clinic before they participated in the campaign, however we did not have access to that information. One study in rural Malawi showed that in a facility offering integrated cervical cancer screening as part of HIV care [38] 73% of women with HIV accessed cervical cancer screening services. While not directly comparable to this setting, this demonstrates the synergistic benefit of caring for both health domains, which could be applied to multi-disease campaigns such as this one.

Among those who were screened, several factors were associated with testing positive for HPV, notably positive HIV status and single marital status. Our study showed that women who tested HPV-positive were more likely to be HIV-positive, a finding that other studies have supported well [39, 40]. As sexual activity and immune-compromise are shared risk factors for HIV and HPV, women living with HIV have higher rates of HPV infection, lower HPV clearance, and a higher incidence of low- and high-grade squamous intraepithelial lesions [39]. Additionally, women who were single (including those who were widowed, divorced, or separated) were more likely to test positive for HPV. Although we did not assess the way being single influences HPV positive status, various explanations are reasonable. Women who are single may have multiple sexual partners and thus be at greater risk. The high rate of HPV positivity among single women may also reflect gender power imbalances with respect to sex and reproductive choice, and that restrict access to financial independence and education. A better understanding of the causes of higher HPV positivity among single women and their perception of HPV risk is needed. Our findings also highlight the need for a health campaign model that integrates cervical cancer screening into the rapid scale-up of HIV testing to reach this high-risk population and have a significant impact.

This study has several limitations that made it challenging to fully determine the acceptability of cervical cancer screening in a multi-disease campaign. First, it used the participants self-reported responses and therefore is subject to response bias. Second, we did not ask participants to report the number of lifetime sexual partners or the last time they received cervical cancer screening, which may have affected their decision to undergo screening. Third, the survey was adapted from our formative work to evaluate the acceptability and feasibility of cervical cancer screening and follow-up care in rural Kenya. Given the large scale of the multi-disease campaign, we shortened the survey to prevent response fatigue, which may have resulted unintentionally in incorporating only the study team’s interest and a priori understanding of why women may or may not choose to be screened. Further, we did not ask questions in the survey to address specific factors that affect screening uptake in multi-disease campaigns other than women’s demographic and clinical characteristics, knowledge, and intention to screen. A qualitative analysis could have been used to explore specific reasons for undergoing screening related to the campaign’s context. Fourth, we did not ask the participants about the reasons they preferred a cervical cancer-specific campaign over a multi-disease campaign. This would have provided critical information about ways to improve the existing multi-disease campaign, particularly the way to tailor it to these individuals’ needs or the way to implement an effective cervical cancer-specific campaign rather than a multi-disease campaign. Fifth, from the study team’s side, we had delays in procuring HPV collection kits, which led the cervical cancer screening portion of the multi-disease campaign to end earlier than intended while other health services at the campaign continued. Therefore, we do not know what the comprehensive outcome of the cervical cancer screening would have been had it been offered to women for the entire duration. Lastly, because this campaign was conducted only once, its long-term effects on the health outcomes and ability to sustain such health services were beyond the scope of the study.

## Conclusion

Our study explores acceptability and uptake of a model of integrated HPV-based cervical cancer screening into multi-disease campaigns offering HIV testing. We also examined the prevalence and predictors of cervical cancer screening and HPV positivity. Although a relatively low cervical cancer screening uptake was observed at the campaign, given the potential benefits of integrating HPV testing into HIV outreach campaigns, facilitators to screening should be examined to develop more effective multi-disease outreach interventions and to optimize the single-visit approach of providing health services. To harness the benefits of multi-disease health campaigns, future integration of cervical cancer screening into multi-disease campaign efforts should target increased male partner involvement in cervical cancer care, focus on addressing a few diseases at multi-disease campaigns based on local epidemiology and national priorities, and adopt a systematic approach to rigorously screen those who are at high risk of developing cervical cancer, such as those who are living with HIV.

## Data Availability

The datasets used and/or analyzed during the current study available from the corresponding author on reasonable request.
